# c-Abl inhibits breast cancer tumorigenesis through reactivation of p53-mediated p21 expression

**DOI:** 10.18632/oncotarget.11909

**Published:** 2016-09-08

**Authors:** Chevaun D. Morrison, Tressa M. Allington, Cheryl L. Thompson, Hannah L. Gilmore, Jenny C. Chang, Ruth A. Keri, William P. Schiemann

**Affiliations:** ^1^ Case Comprehensive Cancer Center, Case Western Reserve University, Cleveland, OH 44106, USA; ^2^ Department of Pharmacology, Anschutz Medical Campus, University of Colorado-Denver, Aurora, CO 80045, USA; ^3^ Department of Nutrition, Case Western Reserve University, Cleveland, OH 44106, USA; ^4^ Department of Pathology, University Hospitals, Case Medical Center and Case Western Reserve University, Cleveland, OH 44106, USA; ^5^ Houston Methodist Research Center, Houston, TX 77030, USA; ^6^ Department of Pharmacology, Case Western Reserve University, Cleveland, OH 44106, USA

**Keywords:** breast cancer, c-Abl, p53, transforming growth factor-β, triple-negative breast cancer

## Abstract

We previously reported that constitutive c-Abl activity (CST-Abl) abrogates the tumorigenicity of triple-negative breast cancer cells through the combined actions of two cellular events: downregulated matrix metalloproteinase (MMP) and upregulated p21^Waf1/Cip1^ expression. We now find decreased c-Abl expression to be significantly associated with diminished relapse-fee survival in breast cancer patients, particularly those exhibiting invasive and basal phenotypes. Moreover, CST-Abl expression enabled 4T1 cells to persist innocuously in the mammary glands of mice, doing so by exhausting their supply of cancer stem cells. Restoring MMP-9 expression and activity in CST-Abl-expressing 4T1 cells failed to rescue their malignant phenotypes; however, rendering these same cells deficient in p21 expression not only delayed their acquisition of senescent phenotypes, but also partially restored their tumorigenicity in mice. Although 4T1 cells lacked detectable expression of p53, those engineered to express CST-Abl exhibited robust production and secretion of TGF-β1 that engendered the reactivated expression of p53. Mechanistically, TGF-β-mediated p53 expression transpired through the combined actions of Smad1/5/8 and Smad2, leading to the dramatic upregulation of p21 and its stimulation of TNBC senescence. Collectively, we identified a novel c-Abl:p53:p21 signaling axis that functions as a powerful suppressor of mammary tumorigenesis and metastatic progression.

## INTRODUCTION

c-Abl is a multifunctional nonreceptor protein tyrosine kinase that regulates the physiology and homeostasis of normal mammary epithelial cells (MECs). However, controversies exist as to the exact role played by c-Abl during the development and progression of solid tumors, including those of the breast. Indeed, several reports have characterized c-Abl as an oncogene that mediates enhanced survival and motility of breast cancer cells [1–9]. In contrast, c-Abl expression and activity are strongly associated with guarding and maintaining the integrity of the genome, doing so through its ability to induce cell cycle arrest and apoptosis in response to DNA damage [10–13]. As such, c-Abl is also implicated as being a powerful suppressor of breast cancer tumorigenicity both *in vitro* and *in vivo* [3, 14–16]. Recent clinical trials designed to assess the effectiveness of the c-Abl inhibitor, Imatinib, which revolutionized the treatment and clinical outcomes for patients with chronic myelogenous leukemia (CML) [17, 18], failed to provide similar benefits in breast cancer patients, many of whom experienced significant toxicity and disease progression in response to Imatinib administration [19–21]. Thus, the functions of c-Abl in normal and malignant MECs are complex and may vary across distinct breast cancer subtypes that possess unique genetic and epigenetic backgrounds.

Transforming growth factor-β (TGF-β) is a multifunctional cytokine that suppresses mammary tumorigenesis by inhibiting cell cycle progression, or by stimulating programmed cell death. Interestingly, late-stage breast cancers, including triple-negative breast cancers (TNBCs), become insensitive to the tumor suppressing activities of TGF-β, and instead readily exhibit epithelial-mesenchymal transition (EMT), invasive, and metastatic phenotypes in response to TGF-β [22]. The acquisition of oncogenic activity by TGF-β largely reflects imbalances between its canonical (*i.e.*, Smad2/3-dependent) and noncanonical (*i.e.*, Smad2/3-independent) signaling systems [23]. Additionally, the presence of a TGF-β gene signature predicts for poor clinical outcomes in TNBCs patients [24], whose tumors lack expression of estrogen receptor-α, progesterone receptor, and ErbB2/HER2, and exhibit high rates of metastasis and disease recurrence [25, 26]. We previously identified c-Abl as a potent suppressor of TNBC tumorigenicity, as well as an inhibitor of oncogenic TGF-β signaling [27]. Indeed, introducing a constitutively-active c-Abl (CST-Abl) mutant into murine TNBCs abrogated their expression of matrix metalloproteinases (MMPs), while simultaneously stimulating that of p21Waf1/Cip1 [27]. Unfortunately, the extent to which either pathway alleviates TNBC tumorigenicity following c-Abl activation remains unknown, as does the role of TGF-β in mediating these events. The goal of this study was to address these important questions and to determine how the c-Abl and TGF-β signaling systems coalesce to elicit senescent phenotypes in TNBCs.

## RESULTS

### Significantly reduced c-Abl expression associates with the progression of human breast cancers

**Figure 1:**
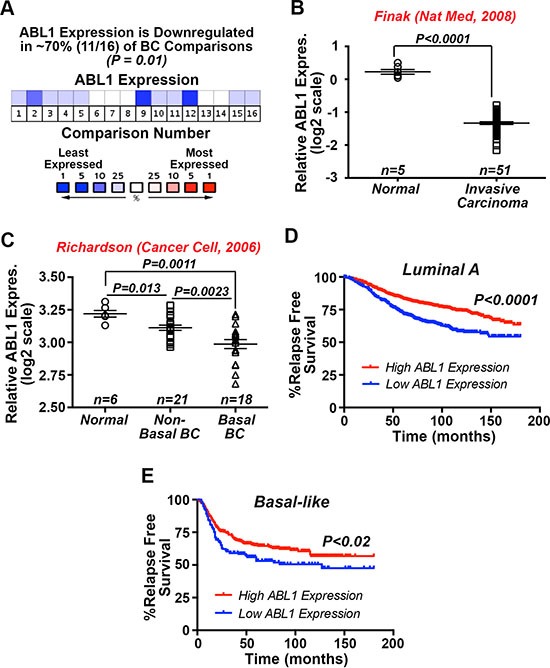
Significantly reduced c-Abl expression associates with the progression of human breast cancers (**A**) Oncomine *in silico* analyses demonstrate that *ABL1* mRNA expression is significantly downregulated in 11 out of 16 study comparisons (~70%; *P* = 0.01), including the Finak [28] (**B**) and Richardson [29] (**C**). (**D** and **E**) Kaplan-Meier plots correlating *ABL1* mRNA expression and the probability of relapse-free survival over 15 years in a cohort of 1678 luminal A (D) and 478 basal-like (E) breast cancer patients.

We previously demonstrated that constitutive c-Abl activity was sufficient to inhibit oncogenic TGF-β signaling and its stimulation of EMT programs in normal and malignant MECs, as well as alleviate TNBC tumor development and metastasis in mice [27]. Additionally, Imatinib administration was ineffective and trended to enhance TNBC tumor growth in preclinical therapy trials in mice, findings reminiscent of those measured in metastatic breast cancer patients treated with Imatinib [27]. The notion that c-Abl functions as a suppressor of mammary tumorigenesis is further supported by the findings presented in Figure 1A, which shows c-Abl transcripts to be significantly downregulated in ~70% of breast cancers. Likewise, interrogating the SAGE Genie tool housed within the Cancer Genome Array Project (http://cgap.nci.nih.gov) showed c-Abl expression to be most dramatically downregulated in breast cancers as compared to tumors arising in other tissues (Supplementary Table S1). Along these lines, Oncomine analyses of the Finak [28] and Richardson [29] datasets demonstrated dramatic reductions of c-Abl expression in invasive carcinomas (Figure 1B), and especially in basal-like breast cancers as compared to their non-basal-like and normal counterparts (Figure 1C). Finally, interrogating the Kaplan-Meier Plotter database (http://kmplot/analysis.com) showed that diminished c-Abl expression is significantly associated with reduced relapse-free survival in luminal A (Figure 1D) and basal-like (Figure 1E) breast cancers. Taken together, these findings reinforce the concept that c-Abl expression and activity suppresses mammary tumorigenesis.

### Enforced c-Abl activation enables TNBCs to persist innocuously in the mammary glands of mice

**Figure 2:**
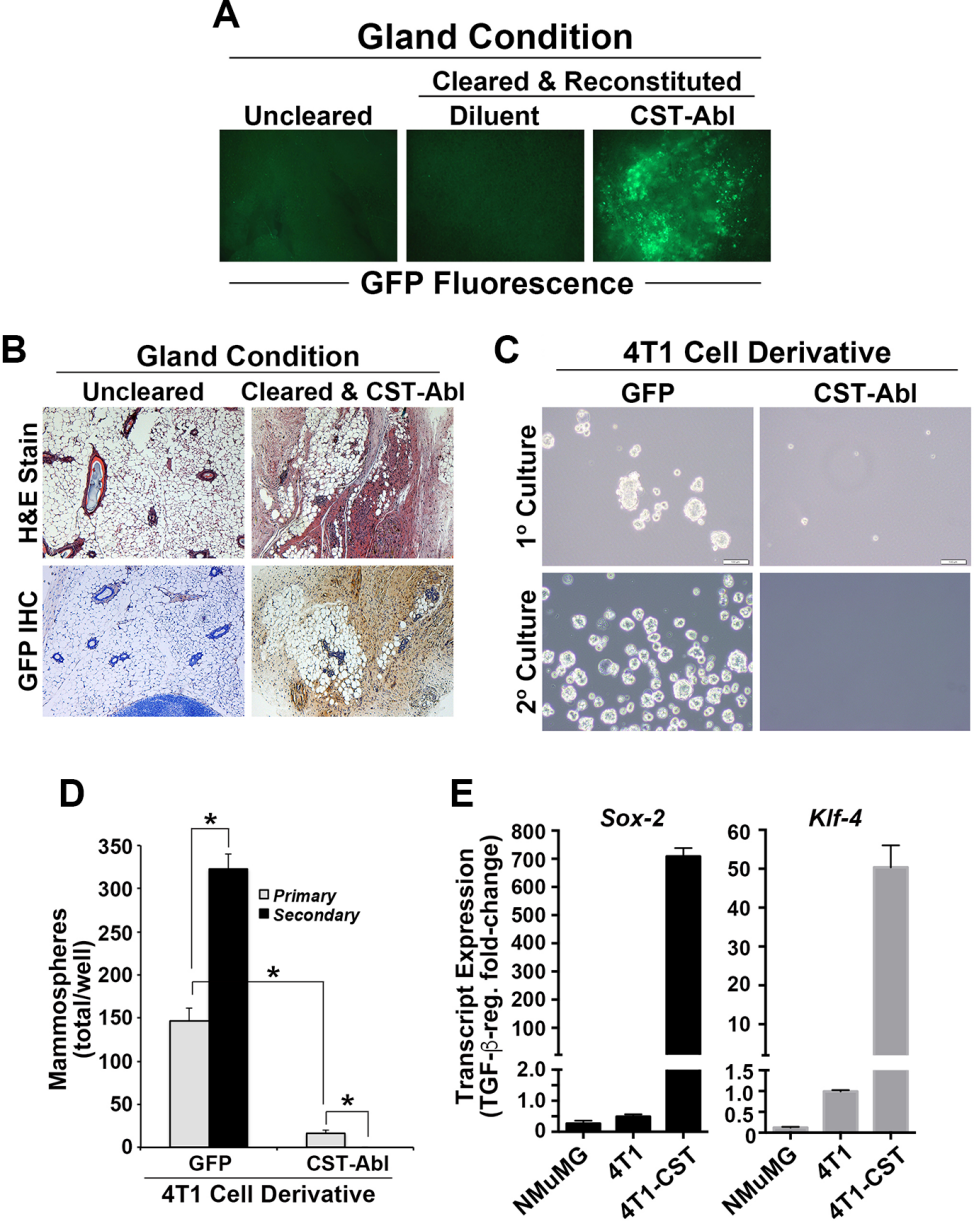
Enforced c-Abl activation enables TNBCs to persist innocuously in the mammary glands of mice (**A**) Whole-mount GFP fluorescence microscopy of mammary glands harvested from right inguinal #4 mammary gland controls, and from cleared left #4 inguinal mammary glands reconstituted with diluent (PBS) or CST-Abl-expressing 4T1 cells as shown. (**B**) H&E *(top)* and anti-GFP IHC *(bottom)* staining of recovered mammary glands demonstrates that CST-Abl-expressing 4T1 cells persisted innocuously in cleared mammary glands. (**C** and **D**) Parental (*i.e.*, GFP) and CST-Abl-expressing 4T1 cells were cultured in ultra-low attachment plates to assess the formation of primary (day 7) and secondary (day 14) mammospheres. Representative photomicrographs from 3 independent experiments are provided (C), as is the mean (± SE) number of mammospheres produced by these analyses (D). *P < 0.05. (**E**) NMuMG, parental 4T1 (*i.e.*, GFP), and CST-Abl-expressing 4T1 cells were incubated in the absence or presence of TGF-β1 (5 ng/ml) for 24 h, at which point alterations in the expression of *Sox-2* and *Klf-4* were determined by semi-quantitative real-time PCR. Data are the mean (± SE) fold-change in gene expression regulated by TGF-β in 3 independent experiments.

Our previous study demonstrated that CST-Abl expression not only inhibited the oncogenic activities of TGF-β, but also induced a mesenchymal-epithelial transition (MET) in 4T1 cells that coincided with their loss of malignant behaviors [27]. Based on these findings, we hypothesized that the ability of CST-Abl to morphologically and phenotypically normalize 4T1 cells would enable them to reconstitute mammary gland morphogenesis following their transplantation into surgically cleared mammary fat pads of virgin female Balb/C mice. As noted previously [27], mice inoculated with parental 4T1 cells (300,000 cells/mouse) rapidly succumbed to lethal tumor burdens within 7 days (*data not shown*), while engrafting those that expressed CST-Abl exhibited no overt signs of mammary tumorigenesis and readily survived for 8 weeks, at which point they were euthanized to facilitate the surgical excision of experimental mammary glands. Contrary to our expectations, the MET program initiated by CST-Abl failed to enable 4T1 cells to reconstitute normal mammary gland morphogenesis in mice, despite the fact that CST-Abl cells readily persisted within the transplanted fat pad as detected by GFP fluorescence and immunohistochemistry (Figure 2A, 2B). The inability of CST-Abl-expressing 4T1 cells to elicit tumor formation (Figure 2; [27]) and mammary gland reconstitution in mice suggested that CST-Abl may inhibit the “stemness” of 4T1 cells. Accordingly, CST-Abl expression abrogated 4T1 cell mammosphere formation (Figure 2C, 2D). Interestingly, induced pluripotent stem cells (iPSCs) can be generated through the simultaneous and enforced expression of Sox2, Klf4, Oct4, and Myc [30], an event coupled to the initiation of MET programs and suppression of EMT stimulated by TGF-β [31]. These events are reminiscent of those attributed to CST-Abl in normal and malignant MECs (Figure 2; [27, 32]). As such, we monitored the effects of CST-Abl in altering Sox2 and Klf4 expression in normal NMuMG and metastatic 4T1 cells. In doing so, we observed Sox2 and Klf4 expression to be significantly downregulated in NMuMG and 4T1 cells following their acquisition of TGF-β-mediated EMT phenotypes (Figure 2E). In stark contrast and consistent with its role as an inducer of MET programs, we observed CST-Abl-expressing 4T1 cells to robustly elevate Sox2 and Klf4 transcript expression when stimulated by TGF-β (Figure 2E). Collectively, these findings and those presented previously [27] demonstrate that the anticancer activities associated with CST-Abl expression are mediated in part *via* the induction of MET programs, which alleviate the cancer-initiating properties of TNBCs.

### Restoring MMP-9 expression and activity in CST-Abl-expressing 4T1 cells fails to rescue their tumorigenicity

TGF-β stimulates breast cancer cells to upregulate MMP expression, particularly that of MMPs 2, 3, and 9 [33], which enhance breast cancer invasion and metastasis [34, 35]. We previously observed *(i)* CST-Abl to prevent TGF-β-mediated expression of MMPs 3, 9, and 13 in 4T1 cells [27], and *(ii)* MMP inhibitors to suppress the growth of 4T1 organoids in 3D-cultures [27, 36]. As such, we hypothesized that reinstating MMP-9 expression and activity in CST-Abl-expressing 4T1 cells would circumvent the antitumor activities mediated by CST-Abl and, consequently, restore their tumorigenicity. To test this hypothesis, we engineered parental and CST-Abl-expressing 4T1 cells to express an autoactivating MMP-9 mutant (*i.e.*, G100L-MMP-9; [37]), as well as rendered parental 4T1 cells deficient in MMP-9 expression by their transduction with a lentiviral shRNA against MMP-9 (Supplementary Figure S1A, 1B). In doing so, we found MMP-9-deficiency to significantly inhibit 4T1 cell proliferation (Supplementary Figure S1C) and invasion to a serum-stimulus (Supplementary Figure S1D); however, this same cellular condition had no effect on the ability of TGF-β to induce 4T1 cell invasion (Supplementary Figure S1D). Interestingly, although the expression of G100L-MMP-9 mutants in CST-Abl-expressing 4T1 cells partially restored their invasiveness to serum, the proteinase activity of this MMP-9 mutant had no effect on TGF-β-stimulated invasion (Supplementary Figure S1E). Along these lines, manipulating the expression and activity of MMP-9 in parental and CST-Abl-expressing 4T1 cells had little-to-no impact on the resultant morphologies exhibited by these cells when propagated in compliant and rigid (*i.e.*, collagen supplementation) 3D-cultures (Supplementary Figure S2). Finally, whereas MMP-9-deficiency significantly enhanced the growth of 4T1 tumors in Balb/C mice, we failed to detect any tumors in mice engrafted with CST-Abl-expressing 4T1 cells that also expressed G100L-MMP-9 mutants (Supplementary Figure S3). Thus, while MMP-9 activity may regulate autocrine TGF-β activity in 4T1 cells (Supplementary Figures S1–S3), the enforced expression and activation of this extracellular protease was unable to circumvent the tumor suppressing functions of CST-Abl in aggressive TNBCs.

### p21^Waf1/Cip1^ expression is required for morphological normalization and cytostasis driven by CST-Abl

**Figure 3:**
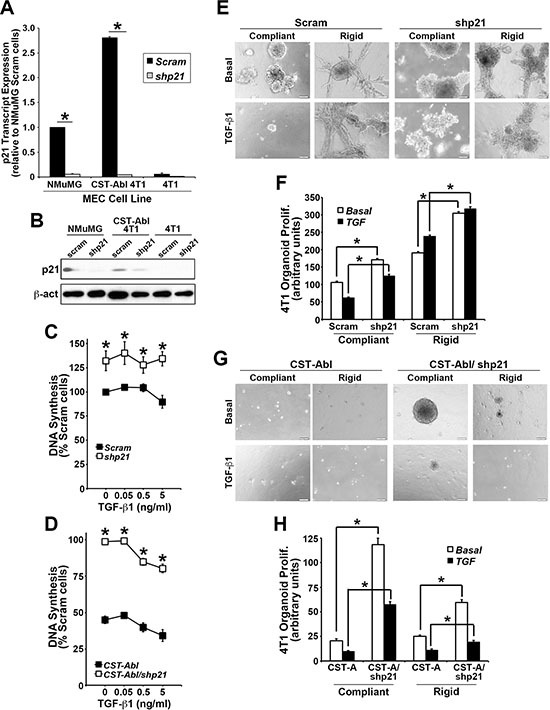
p21 expression is required for morphological normalization and cytostasis driven by CST-Abl (**A** and **B**) Endogenous and genetically manipulated expression of p21 (*i.e.*, shp21) in NMuMG, 4T1, and CST-Abl-expressing 4T1 cells was monitored by semi-quantitative real-time PCR (A), and by immunoblotting (B). Data are the mean (± SE) of 3 independent experiments. *P < 0.05. (**C** and **D**) Alterations in DNA synthesis regulated by TGF-β1 (0**→**5 ng/ml) was determined by [^3^H]thymidine incorporation assays. Data are the mean (± SE) of 3 independent experiments as indicated. (**E** and **F**) 4T1 derivatives employed in Panels C and D were propagated in the absence or presence of TGF-β1 (5 ng/ml) for 7 days in either compliant or rigid 3D-cultures as indicated. Alterations in organoid growth regulated by TGF-β1 (0**→**5 ng/ml) was determined by Image J analyses. Data are the mean (± SE) of 4 independent experiments as indicated.

p21^Waf1/Cip1^ is a potent tumor suppressor that prevents uncontrolled cell proliferation by inducing cell cycle arrest, and stimulating cell differentiation. p21 expression is regulated by various factors and pathways, including several anticancer agents (*e.g.*, statins and HDAC inhibitors), DNA damage, p53 and p73, oxidative stress, cytokines and growth factors, and TGF-β [38–40]. Interestingly, p21 expression has also been linked to the acquisition of senescence phenotypes in breast cancer cells [41]. Because p21 is a major downstream effector of c-Abl in 4T1 cells [27], we next addressed the role of p21 in mediating the anticancer activities CST-Abl in normal and malignant MECs. In doing so, we transduced normal NMuMG, parental 4T1, and CST-Abl-expressing 4T1 cells with lentiviral shRNA particles to deplete their expression of p21 mRNA (Figure 3A) and protein (Figure 3B). Functionally, p21-deficiency markedly increased the magnitude of DNA synthesis in all three cell lines when propagated in 2D-cultures (Figure 3C, 3D), as well as uncoupled TGF-β from regulating cytostasis in NMuMG cells (Supplementary Figure S4A). In addition, we previously noted that the enforced expression of CST-Abl in NMuMG cells arrests their proliferation after 2–4 divisions in 3D-cultures, and that parental and CST-Abl-expressing NMuMG cells apoptose when stimulated by TGF-β in rigid 3D-cultures [27]. Supplementary Figure S4B, S4C shows that p21-deficiency also significantly enhanced the basal proliferation rate of NMuMG cells propagated under either compliant or rigid conditions, and that p21-deficient NMuMG cells were strikingly resistant to TGF-β-mediated apoptosis under these same cellular conditions. The enhanced resistance to apoptosis mediated by p21-deficiency did not appear to reflect differences in NMuMG cell “stemness” as parental and p21-deficient NMuMG cells exhibited similar defects in mammosphere maintenance (Supplementary Figure S5A). Thus, these findings suggest that the loss of p21 expression may confer normal MECs the ability to survive the tumor suppressing activities of TGF-β, thereby contributing to their eventual transformation.

Although 4T1 cells are insensitive to the anti-proliferative activities of TGF-β in traditional 2D-cultures (Figure 3C), we previously demonstrated that propagating these TNBCs in compliant 3D-cultures partially restores their cytostatic response to TGF-β, an event that is lost by culturing 4T1 cells in mechanically rigid 3D-cultures [27, 42]. Morphologically, CST-Abl-expressing 4T1 cells form small spherical and partially hollowed organoids in compliant 3D-cultures, which contrasted sharply with the large irregular organoid structures formed by their parental counterparts under identical culture conditions [27]. As expected, TGF-β inhibited the growth of 4T1 organoids in compliant 3D-cultures, but readily stimulated their proliferation in rigid 3D-cultures (Figure 3E, 3F). Interestingly, p21-deficiency significantly enhanced the proliferation of parental and CST-Abl-expressing 4T1 organoids in both compliant and rigid 3D-cultures (Figure 3E–3H). As noted previously [27], CST-Abl expression restores the cytostatic activities of TGF-β under both compliant and rigid 3D-culture conditions, a reaction that now appears to be partially dependent upon p21 expression (Figure 3C, 3D). Similar to NMuMG cells, the discrepancies in 4T1 cell behavior resulting from p21-deficiency did not reflect differences in the ability of parental and p21-deficient 4T1 cells to form mammospheres (Supplementary Figure S5B). Interestingly, introducing CST-Abl into 4T1 cells dramatically inhibited their ability to produce and maintain mammospheres when serially passaged. Moreover, Supplementary Figure S5B also shows that p21-deficient CST-Abl-expressing 4T1 cells initially enhanced their production of primary mammospheres, which were subsequently exhausted through serial passages. Collectively, these findings indicate that the induction of p21 expression by CST-Abl (Figure 3A; [27]) plays an essential role in suppressing TNBC growth, doing so in part by altering their self-renewal capacity.

### CST-Abl governs the coupling of TGF-β to the activation of Smad2/3 and Smad1/5/8 in 4T1 cells

**Figure 4:**
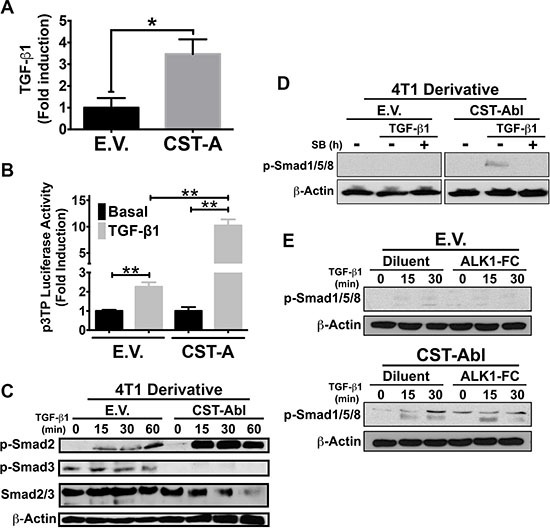
CST-Abl governs the coupling of TGF-β to the activation of Smad2/3 and Smad1/5/8 in 4T1 cells (**A**) CST-Abl expression induces the production and secretion of TGF-β1 from 4T1 cells as measured by ELISA assays. Data are the mean (± SE) fold-induction of TGF-β1 in 3 independent experiments. *P < 0.05. (**B**) Parental (E.V.) and CST-Aβl (CST-A)-expressing 4T1 cells were transiently transfected with p3TP-luciferase and with pCMV-β-gal, and subsequently were stimulated with TGF-β1 (5 ng/ml). Data are the mean (± SE) fold-induction of luciferase activity normalized to that of β-gal in 3 independent experiments. **P < 0.01. **(C**) Parental (E.V.) and CST-Aβl-expressing 4T1 cells were stimulated with TGF-β1 (5 ng/ml) as indicated in the absence or presence of the TβRI inhibitor, SB431542 (10 μM; Panel **D**) or the BMP antagonist, ALK1-Fc (200 ng/ml; Panel **E**). Afterward, the activation status of Smad2, Smad3, and Smad1/5/8 were monitored by immunoblotting as indicated. Differences in protein loading were monitored by reprobing stripped membranes with antibodies against total Smad2/3 and β-actin as indicated. Data are representative images of 4 (C), 2 (D), or 2 (E) independent experiments, respectively.

Findings presented in Figures 2 and 3 suggest that CST-Abl inhibits oncogenic TGF-β signaling; they also point to the intriguing possibility that this event may in fact be reliant upon signaling inputs derived from TGF-β receptors. In addressing this hypothesis, we initially measured the concentrations of TGF-β1 produced by parental and CST-Abl-expressing 4T1 cells. As shown in Figure 4A, CST-Abl expression significantly increased the production and secretion of TGF-β1, leading to augmented TGF-β signaling as well (Figure 4B). Interestingly, although TGF-β readily stimulated Smad2 and Smad3 in 4T1 cells, we only detected the activation of Smad2 in CST-Abl-expressing 4T1 cells treated with TGF-β (Figure 4C). In light of recent findings demonstrating crosstalk between BMP- and TGF-β-regulated Smads [43–45], we next determined whether CST-Abl expression influenced the coupling of TGF-β to classical BMP-regulated responses. Interestingly, although TGF-β could indeed stimulate the phosphorylation of Smad1/5/8, this event only transpired in CST-Abl-expressing 4T1 cells and was readily inhibited by administration of the TβR-1/ALK-5 inhibitor, SB431542 (Figure 4D). Likewise, the ability of TGF-β to activate a BMP-responsive reporter gene (*i.e.*, BRE-luciferase) was also specific to CST-Abl-expressing 4T1 cells *(data not shown)*. Finally, we asked whether the TGF-β-dependent increase in Smad1/5/8 phosphorylation in CST-Abl expressing 4T1 cells was dependent upon the activity of the BMPR1A. Figure 4E shows that TGF-β stimulation of Smad1/5/8 phosphorylation was unaffected by administration of the BMP antagonist, BMPR1A (ALK1-FC), which robustly inhibited the coupling of BMP2 to Smad1/5/8 phosphorylation and BRE-driven luciferase expression *(data not shown).* Collectively, these findings demonstrate that the coupling of TGF-β to Smad1/5/8 was dependent upon the activities of c-Abl and TβRI, and conversely, were independent of BMP type I receptors.

### p21-deficiency partially restores the tumorigenicity of CST-Abl-expressing 4T1 cells

**Figure 5:**
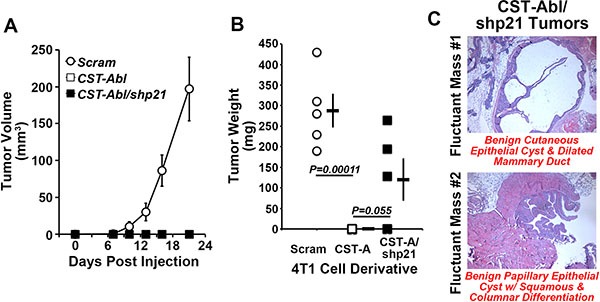
p21-deficiency partially restores the tumorigenicity of CST-Abl-expressing 4T1 cells (**A** and **B**) Parental (*i.e.*, Scram), CST-Abl-, and CST-Abl/shp21-expressing 4T1 cells were engrafted onto the mammary fat pads of Balb/C mice. The resultant tumor growth was measured by digital calipers (A), and by excising and weighing the tumors at the time of necropsy (B). Data are the mean (± SE) tumor volumes (A) and weights (B). (**C**) H&E staining of paraffin-embedded slices of mammary glands harvested from p21-deficient CST-Abl-expressing 4T1 tumors.

Based on the ability of p21-deficiency to *(i)* enhance normal and malignant MEC proliferation and survival in 2D- and 3D-culture systems, and *(ii)* partially circumvent the anticancer activities of CST-Abl in 4T1 organoids (Figure 3), we next sought to determine whether p21-deficiency could restore the tumorigenicity of CST-Abl-expressing 4T1 cells following their engraftment onto the mammary fat pads of syngeneic Balb/C mice. Figure 5 shows that rendering CST-Abl-expressing 4T1 cells deficient in p21 expression failed to restore overt signs of disease to these mice as gauged by regular palpation and caliper measurements, even out to 59 days post-inoculation when the mice were sacrificed and their mammary glands were recovered for pathological analyses (Figure 5A, 5B). Interestingly, we identified small fluctuant masses in the glands of 3 of 5 mice injected with p21-deficient CST-Abl-expressing 4T1 cells (Figure 5C), while no such abnormalities were observed in animals inoculated with their p21-expressing counterparts (*data not shown*). Histopathological analysis identified one mass as being a benign cutaneous cyst of epithelial origin associated with ductal dilation, while the second mass was determined to be a benign papillary epithelial tumor associated with squamous and columnar cell differentiation (Figure 5C). Thus, while depleting p21 expression in CST-Abl-expressing 4T1 cells results in the formation of benign cystic mammary lesions, this event nevertheless remained insufficient to fully circumvent the anticancer activities of activated c-Abl. Collectively, these findings suggest that elevated p21 expression is likely one component of a large scale reprogramming effort elicited by CST-Abl-expression in late-stage TNBC cells.

### CST-Abl expression abrogates 4T1 tumorigenicity by inducing a p21-dependent senescence

**Figure 6:**
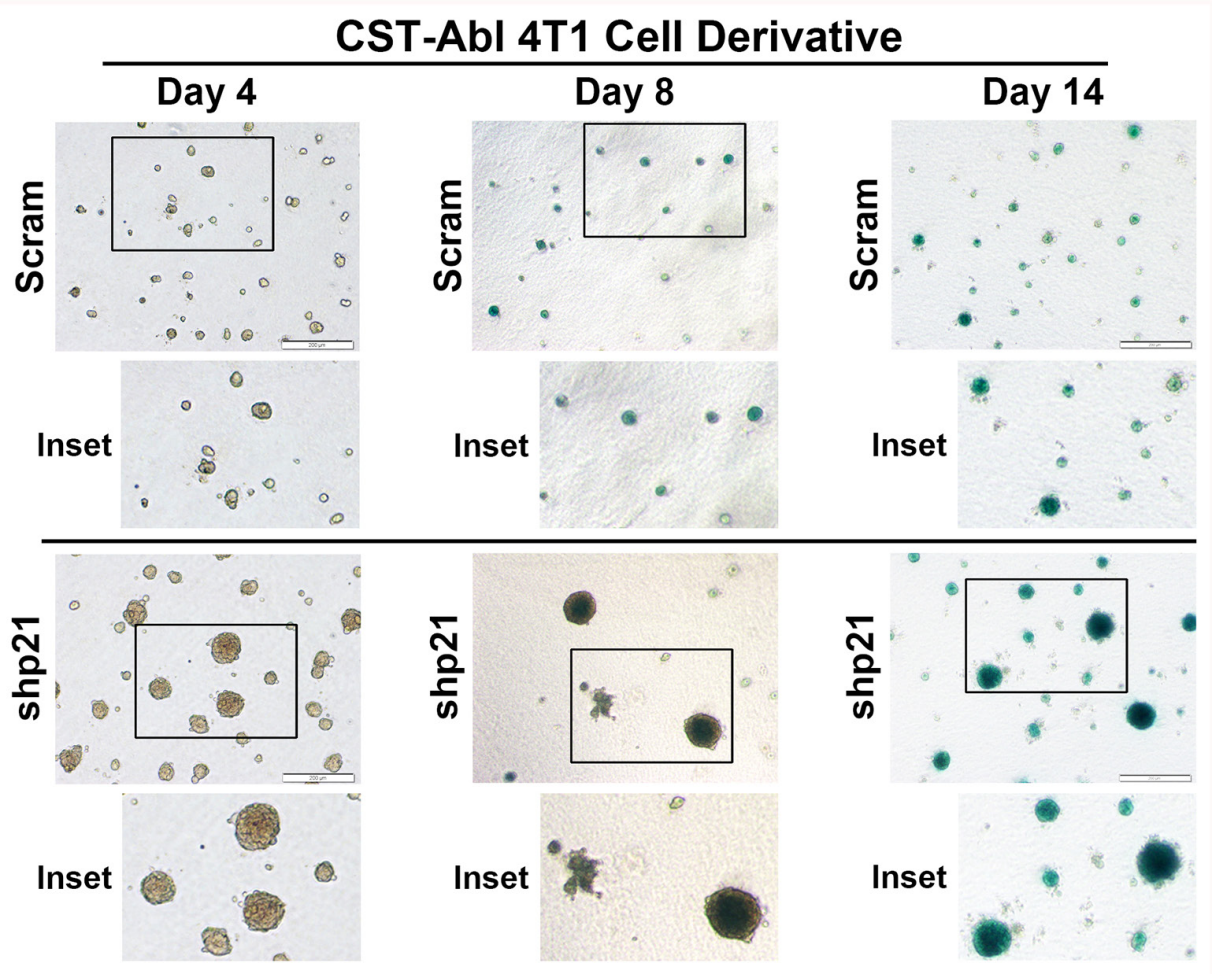
CST-Abl expression abrogates 4T1 tumorigenicity by inducing a p21-dependent senescence CST-Abl-expressing 4T1 cells were transduced with scrambled (*i.e.*, Scram) or p21 shRNA lentiviral particles. Afterward, the 4T1 derivatives were propagated in compliant 3D-cultures for 4–14 days, at which point they were fixed and stained for β-galactosidase expression. Photomicrographs are representative of 2 independent experiments.

Following their expression of CST-Abl, 4T1 cells form extremely small and perfectly spherical organoids when propagated in compliant 3D-cultures (Figure 3G; [27]). Indeed, organoids formed by CST-Abl-expressing 4T1 cells appeared to be stable and could remain in this state for extended culture periods (*e.g.*, > 1 month; *data not shown*), which in many respects recapitulated their inability to form tumors in mice despite their protracted residence within the mammary gland (Figure 2). The expression of p21 has been linked to senescence phenotypes [46–48], and as such, we speculated that one mechanism whereby CST-Abl suppresses TNBC tumorigenicity is through a p21-dependent senescent reaction. To test this supposition, we propagated control (*i.e.*, scrambled shRNA) or p21-deficient CST-Abl-expressing 4T1 cells in compliant 3D-cultures over a span of 14 days, at which point the resulting organoids were stained for β-galactosidase-mediated cleavage of X-gal (5-bromo-3-indolyl-β-D-galactorpyranoside) to monitor the extent of cellular senescence. Figure 6 shows that control CST-Abl-expressing 4T1 organoids failed to grow significantly beyond day 4 and remained perfectly spherical throughout the 14-day time-course. X-gal staining of fixed organoids indicated that control CST-Abl-expressing 4T1 cells expressed robust quantities of senescence-associated β-galactocidase by day 8, demonstrating that these TNBCs underwent senescence between 4–8 days in compliant 3D-cultures (Figure 6). In stark contrast, organoids formed by p21-deficient CST-Abl-expressing 4T1 cells were significantly larger and displayed more heterogeneous morphologies, as well as exhibited delayed acquisition of senescent phenotypes as compared to their p21-expressing counterparts (Figure 6). Moreover, the rate at which CST-Abl-expressing 4T1 derivatives underwent senescence was unaffected by alterations in microenvironmental compliance (*i.e.*, collagen supplementation; *data not* shown), nor was the expression of senescence-associated β-galactosidase activity ever detected in parental 4T1 cells cultured under identical conditions (*data not shown*). Collectively, these findings indicate that p21-deficiency can delay, but not prevent, the ability of CST-Abl to induce senescence in late-stage TNBCs, results that are consistent with the development of benign cystic lesions by p21-deficient CST-Abl-expressing 4T1 cells in mice (Figure 5C).

### c-Abl induces p21 expression *via* a TGF-β1-dependent reactivation of p53 expression in 4T1 cells

**Figure 7:**
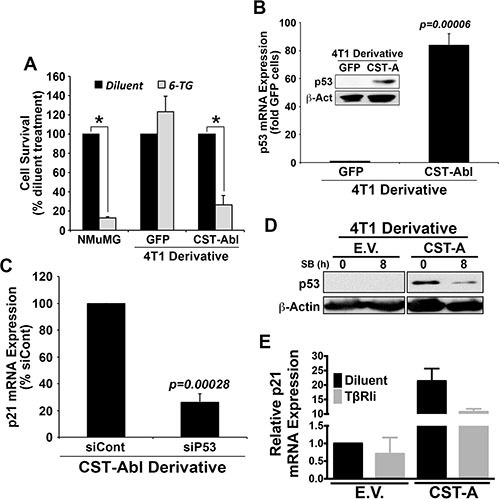
c-Abl induces p21 expression *via* a TGF-β1 dependent reactivation of p53 expression in 4T1 cells (**A**) NMuMG, parental 4T1 (*i.e.*, GFP), and CST-Abl-expressing 4T1 cells were incubated with 6-thioguanine (6-TG) for 72 h. Data are the mean (± SE) percentage of surviving cells observed in 3 independent experiments. *P < 0.05. (**B**) Expression of p53 mRNA and protein (inset) in parental (*i.e.*, GFP) and CST-Abl-expressing 4T1 cells. Data are the mean (± SE) of 3 independent experiments. (**C**) CST-Abl-expressing 4T1 cells were transiently transfected with nontargeting (*i.e.*, siCont) or p53-targeting siRNA. Differences in p21 expression were determined on day 5 by semi-quantitative real-time PCR. Data are the mean (± SE) of 3 independent experiments. (**D**) Parental (E.V.) and CST-Abl-expressing (CST-A) 4T1 cells were incubated for 8 h in the absence or presence of SB431542 (10 μM) as indicated, at which point differences in p53 expression were determined by immunoblotting. Data are representative of 2 independent experiments. (**E**) Parental (E.V.) and CST-Abl-expressing (CST-A) 4T1 cells were grown in compliant 3D-cultures for 60 h in the absence or presence of SB431542 (10 μM). Afterward, p21 mRNA expression was determined by semi-quantitative real-time PCR. Data are the mean (± SD) of 2 independent experiments.

The aforementioned findings led us to speculate the existence of an anticancer signaling axis comprised of c-Abl and p21 in TNBCs. Indeed, c-Abl is well known for its ability to regulate apoptosis and cell cycle progression by modulating the activity of p53 family members in response to DNA damage [12, 49] either through a direct induction of target gene expression (*e.g.*, p73), or indirectly through c-Abl-mediated phosphorylation of MDM2, which releases p53 and facilitates its activation of target gene expression [50–52]. As expected, treating NMuMG cells with the DNA damage-inducing agent, 6-thioguanine (6-TG) resulted in massive cell death (Figure 7A), an event that contrasted sharply with the complete insensitivity of 4T1 cells to undergo apoptosis in response to 6-TG (Figure 7A). Interestingly, enforced CST-Abl expression sensitized 4T1 cells to DNA damage-induced cell death elicited by 6-TG, suggesting that c-Abl expression enhances the anticancer activities of DNA damaging agents in TNBCs. Treatment with Nutlin-3, which inhibits the interaction between MDM2 and p53 or p73, had no effect on the sensitivity and survival of 4T1 cells to 6-TG *(data not shown)*. Mechanistically, parental 4T1 cells were observed to be devoid of p53 expression, while their CST-Abl-expressing counterparts were found to harbor robust quantities of p53 mRNA and protein (Figure 7B). In light of the ability of p53 to induce p21 expression, we reasoned that CST-Abl might couple to p21 expression indirectly *via* activation of p53 expression. To test this hypothesis, we transiently transfected CST-Abl-expressing 4T1 cells with control (*i.e.*, nontargeting) or p53-targeting siRNAs, of which the latter experimental condition dramatically downregulated p21 expression in CST-Abl-expressing 4T1 cells (Figure 7C). Moreover, treating CST-Abl-expressing 4T1 cells with the TβRI inhibitor, SB431542 significantly reduced their expression of p53 (Figure 7D) and p21 (Figure 7E), suggesting that the reactivation of the p53 and p21 by c-Abl is dependent upon TGF-β receptors. Taken together, these findings identify a novel c-Abl-dependent TGF-β →p53 →p21 signaling axis that inhibits the tumorigenicity of TNBCs by inducing their acquisition of senescent phenotypes.

### c-Abl and p53 are discordantly expressed in triple negative breast cancer

**Figure 8:**
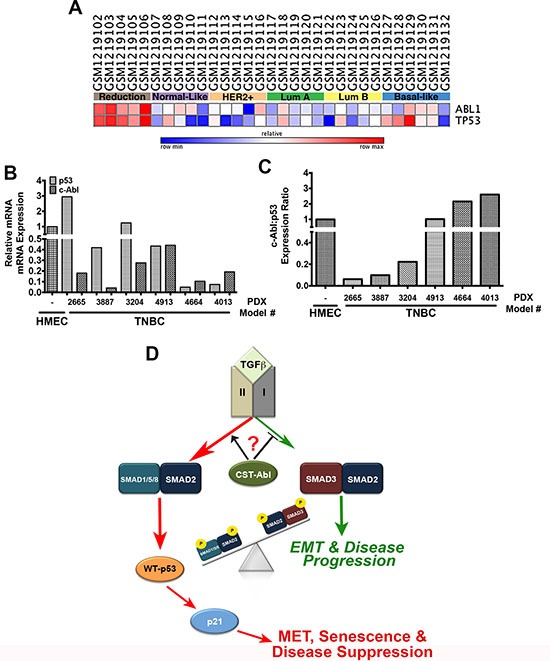
c-Abl and p53 are discordantly expressed in breast cancer (**A**) c-Abl and p53 mRNA expression in the indicated NCBI geo datasets was analyzed by GENE-E (www.broadinstitute.org/cancer/software/GENE-E). (**B**) c-Abl and p53 mRNA expression was determined by semi-quantitative real-time PCR in normal HMEC organoids and TNBC PDX models. (**C**) The ratio of c-Abl:p53 expression determined in *Panels C* and *D* indicate that these tumor suppressors are discordantly expressed TNBC tumors. (**D**) Representative model highlighting the necessity of c-Abl expression and activity for the induction of MET reprogramming and TNBC tumor suppression, which transpires through an imbalance in activation of pSmad2:pSmad1/5/8 relative to that of pSmad3 in response to increased autocrine TGF-β1 signaling. Questions remain as to the precise role activated c-Abl plays in driving the imbalances in Smad activation by TGF-β.

Lastly, we examined the expression patterns of c-Abl and p53 across normal and malignant breast tissues. In doing so, we observed a strong concordance for both tumor suppressors to be expressed in normal breast tissues obtained from mammoplastic reduction surgeries, and in luminal A and B breast cancer subtypes. In stark contrast, c-Abl and p53 exhibited highly discordant expression patterns in basal-like breast cancers (Figure 8A). c-Abl expression was also dramatically downregulated in a panel of TNBC PDX tumors as compared to normal human mammary epithelial cells (HMECs) isolated from mammoplastic reduction surgery (Figure 8B). Interestingly, TNBC PDX tumors exhibited a diverse range of p53 expression (Figure 8B) that was inversely related to the expression of c-Abl in identical PDX tissues (Figure 8C). Finally, we assessed the expression of c-Abl and p53 by immunostaining adjacent slices of a breast tissue microarray (TMA) that contained 157 breast tissues and spanned all major histological subtypes (Supplementary Figure S6A; [53]). These analyses determined that robust c-Abl expression trended to associate with improved overall patient survival (Supplementary Figure S6B); however, when we further discriminated for the expression status of p53, we found that wild-type p53 expression combined with robust expression of c-Abl trended to provide better overall survival as compared to tumors that housed high expression of c-Abl and mutant p53 (Supplementary Figure S6C). Thus, the anticancer activities attributed to dual c-Abl and p53 expression appear to be more protective than those mediated by the singular stratification of c-Abl in human breast cancers. Collectively, these findings indicate that the levels of p53 expression and its mutational status are critical determinants of the tumor suppressing activities of c-Abl in human breast cancers.

## DISCUSSION

Our previous findings [27] and those presented herein clearly show that the anticancer activities of CST-Abl are robust, multifactorial, and dependent upon the expression and mutational status of p53. The essential genome guarding functions of c-Abl in regulating DNA damage responses *via* p53 and p73 are well-established [54, 55]; however, to our knowledge, our study is the first to establish the importance of an autocrine c-Abl →TGF-β**→**TβRI→Smad1/5/8:Smad2→p53→p21 signaling axis in dictating MEC morphology and suppressing tumorigenesis. The importance of p53 in mediating the tumor suppressing activities of c-Abl cannot be overstated, as p53 is an essential tumor suppressor and guardian of the genome; it also serves in repressing the proliferation of mammary stem/progenitor cells [56]. Our findings indicate that c-Abl-mediated reactivation of p53 suppresses CSC renewal and expansion, which renders 4T1 cells innocuous when engrafted into mice. Conversely, mutant p53 expression trended to reverse the overall survival benefit provided to breast cancer patients whose tumors retain high expression of c-Abl (Supplementary Figure S6), a finding that is highly significant in light of the fact that mutant p53 can promote oncogenic TGF-β signaling [57]. Moreover, wild-type p53 and TGF-β cooperate in stimulating the expression of their downstream effectors, particularly p21, whereas mutant p53 antagonizes these events in part by downregulating the expression of TβRII [58]. Thus, it is tempting to speculate that mutant p53 underlies the initiation of oncogenic TGF-β-signaling and its ability to circumvent the tumor suppressing activities of c-Abl observed in our patient cohort. As such, the functional interaction between mutant p53 and c-Abl requires further evaluation related to their relevance in regulating breast cancer progression.

Although the mechanism whereby 4T1 cells lose p53 expression remains unknown, our findings indicate that oncogenic TGF-β signaling plays an intimate role in this process. Indeed, we observed enforced c-Abl activity to drive p53 expression *via* TGF-β stimulation of Smad1/5/8 and Smad2. Precisely how c-Abl rewires the TGF-β pathway and its reactivation of p53 requires further investigation; however; c-Abl can phosphorylate Mdm2 at Tyr394, which stabilizes and activates p53 [51], as well as promotes its nuclear accumulation following DNA damage [59]. Accordingly, we observed 4T1 cells to elevate their expression of Mdm2 and suppress that of p53 in response to TGF-β, events that were readily reversed in CST-Abl-expressing 4T1 cells *(data not shown).* Interestingly, when activated by genotoxic stress, ATM can stimulate Smad1 and promote its direct interaction with p53, thereby protecting p53 from Mdm2-mediated ubiquitination and degradation [60]. Thus, the loss of c-Abl expression in TNBCs may disinhibit Mdm2 activity, leading to the functional inactivation of p53 and the initiation of oncogenic signaling by TGF-β. Along these lines, we observed c-Abl and p53 to be readily co-expressed in normal human breast tissues, an event that rarely occurred in their malignant counterparts. The discordance in c-Abl and p53 expression patterns may explain the lack of efficacy provided by Imatinib to patients harboring cancers of the breast [19–21], thyroid [61], and prostate [62–64], all of which represent tissues that normally co-express c-Abl and p53. Interestingly, the expression levels of c-Abl and p53 are inversely correlated in bone marrow, a tissue wherein c-Abl acquires oncogenic activity during the initiation of CML [17, 18]. Thus, elucidating and ultimately inactivating the mechanisms operant in downregulating c-Abl expression and activity may provide a novel therapeutic strategy to restore p53 expression in distinct subtypes of breast cancer.

Lastly, our findings suggest the intriguing possibility that the anticancer activities of c-Abl occur predominantly in a cell-autonomous manner, and that c-Abl dictates the coupling of TGF-β receptors to various Smad family members. For instance, p21-deficiency dramatically increased the proliferative activity of NMuMG and 4T1 cells in both 2D- and 3D-cultures. Interestingly, TGF-β induces extensive apoptosis in NMuMG cells cultured in mechanically rigid microenvironments, an event that was noticeably absent in normal and malignant MECs rendered deficient in p21 expression. These findings reinforce the important links between mechanotransduction and TGF-β signaling [27, 42, 65–68] and suggest that the uncoupling of c-Abl to p21 expression represents an early event in mammary tumorigenesis, perhaps serving to enhance the dedifferentiation, expansion, and self-renewal properties of breast CSCs. Indeed, when stimulated with TGF-β, CST-Abl-expressing 4T1 cells expressed robust levels of the MET-associated transcription factors, Sox2 and KLF-4. Moreover, enforced expression of KLF-4 in 4T1 cells suppressed their tumorigenicity and metastasis, doing so by inhibiting Snail expression and its induction of EMT programs [69]. Likewise, KLF-4 induces p21 expression in a context-specific manner, such that inactivation of p21 converts KLF-4 from a tumor suppressor to a tumor promoter [70, 71]. It is interesting to note that KLF-4 also interacts physically with Smad1 [72], a BMP-regulated Smad whose unusual activation by TGF-β we show is governed by CST-Abl. BMPs belong to the TGF-β superfamily and have been shown to inhibit TGF-β-dependent EMT and the self-renewal properties of CSCs; they also induce dormant states in disseminated breast cancer cells, thereby preventing their metastatic outgrowth [73–75]. Accumulating evidence also indicates that TGF-β can engage and activate Smad1/5/8 during tumor progression [43–45]. Somewhat surprisingly, we found c-Abl to serve as a molecular switch that couples Smad1/5/8 signaling to that of Smad2, an event that transpires in the absence of Smad3 and its propensity to enhance tumorigenesis. Likewise, phosphorylated Smad3 can form a complex with Smads 1 and 5 to inhibit BMP-dependent signaling, thereby preventing the ability of TGF-β to induce the expression of BMP-responsive genes [76]. Previous studies have also found that *(i)* c-Abl phosphorylates the type I BMP receptor (BMPR1A) to differentially regulate canonical and noncanonical BMP pathways to promote osteoblast differentiation [77], and *(ii)* BMP-2-induced c-Abl kinase activity is required for the osteoclast formation through coordinate phosphorylation of BMPR1A and Smad1/5/8 [78]. Thus, by preventing Smad3 activation by TGF-β, c-Abl expression and activity favor the induction of MET and senescence programs *via* Smad1/5/8- and Smad2-dependent transcriptional programs (Figure 8D.) Future studies need to assess whether the pro-BMP activities of c-Abl transpire through alterations in either the production of BMP ligands (*i.e.*, increased) or antagonists (*i.e.*, decreased), as well as determine the extent which c-Abl orchestrates TβRI-mediated activation of Smad1/5/8 in TNBCs.

## MATERIALS AND METHODS

### Cell lines and retroviral vectors

Normal murine NMuMG mammary epithelial cells and metastatic murine 4T1 breast cancer cells were obtained from ATCC (Manassas, VA) and cultured as described previously [27]. A retroviral vector (pMSCV-hygromycin) that encoded murine CST-Abl (type IV) was generously provided by Dr. Tony Hunter (Salk Institute, La Jolla, CA; [79–81] and used to produce polyclonal populations of 4T1 cell lines that stably expressed either empty vector (*i.e.*, parental) or CST-Abl as previously described [27]. Murine p21 expression was functionally disrupted by transducing NMuMG and 4T1 derivatives with lentiviral particles that encoded for shRNA against p21 (cat# RMM4534-NM_001111099, Open Biosystems, Huntsville, AL) as described previously [27]. The extent of p21-deficiency was determined by real-time PCR and immunoblotting as described below.

### TGF-β1 Enzyme-linked immunosorbent assay (ELISA)

The production and secretion of TGF-β1 from 4T1 cell derivatives was measured using the mouse TGF-β1 Quantikine ELISA kit (MB100B; R&D Systems, Minneapolis, MN) according to the manufacturer's instructions. Briefly, 4T1 cell derivatives were cultured for 24 hr in serum-free DMEM, at which point the conditioned-media was collected, centrifuged at 500 g for 3 min, and acidified to measure total TGF-β1 concentrations (*i.e.*, latent and active). The protein content of the remaining adherent 4T1 derivatives was determined and used to normalize TGF-β1 concentrations (*i.e.*, TGF-β1 pg/mg protein).

### Mammary gland reconstitution, imaging, and immunohistochemistry

Mammary gland reconstitution analyses were performed as previously described [82]. Briefly, 3-week-old female Balb/C mice (6 mice/cell line) were anesthetized under isofluorane gas to permit the surgical removal of the left inguinal #4 mammary gland and lymph node, except for a small dorsal segment of the fat pad that was reserved and injected with either parental (*i.e.*, GFP-expressing) or CST-Abl-expressing 4T1 cells (300,000 cells/injection). At 8-weeks post-surgery, the mice were sacrificed and the manipulated glands were removed and whole-mounted between slides to immediately image GFP expression on a Leica fluorescent microscope. The right inguinal #4 mammary glands were also harvested at this time and served as positive controls for mammary gland development, while cleared mammary fat pads injected with PBS served as negative controls for mammary gland reconstitution. Following GFP imaging, the whole glands were formalin fixed and paraffin embedded (IHCTech, Aurora, CO) prior to assessing overall glandular morphology and development by hematoxylin and eosin (H&E) staining, and by immunohistochemistry for GFP and β-galactosidase expression (IHCTech, Aurora, CO).

### Mammosphere assays

Mammosphere assays were executed as described previously [83]. Briefly, single cell suspensions of NMuMG or 4T1 derivatives were prepared and plated (10,000 cells/well) in 6-well, ultra-low attachment plates (Corning, Corning, NY). The cultures were fed every 3–4 days with serum-free DMEM (Invitrogen, Carlsbad, CA) supplemented with bFGF (20 ng/ml; Invitrogen), EGF (20 ng/ml; Invitrogen), B27 (Life Technologies, Grand Island, NY), and heparin (4 mg/ml; Sigma, St. Louis, MO), and the resulting mammospheres were enumerated on day 7 by light microscopy. Afterward, the primary mammospheres were collected by gentle centrifugation, and subsequently were disrupted by trypsinization and serially passaged at a density of 1,000 cells/well for an additional 7 days to assess the formation of secondary mammospheres.

### Semi-quantitative real-time PCR

4T1 (250,000 cells/well) or NMuMG (350,000 cells/well) cells were cultured overnight in 6-well plates. The following morning, the cells were fed with fresh media and immediately incubated in the absence or presence of TGF-β1 (5 ng/ml) for 48 hr at 37°C. Afterward, total RNA was harvested and employed to monitor the expression of *Sox-2, Klf-4*, and *p21* by semi-quantitative real-time PCR as described [27]. Total RNA was also harvested from frozen sections of low-passage human breast PDX tumors [84] and used to monitor the expression of *c-Abl* and *p53* by semi-quantitative real-time PCR. In all cases, differences in RNA concentration for individual genes were normalized to their corresponding RNA signals for GAPDH. The oligonucleotides primer pairs used are provided in Supplementary Table S2.

### Cell biological assays and immunoblotting

The ability of TGF-β1 (0**→**5 ng/ml) to impact DNA synthesis in NMuMG and 4T1 derivatives was determined by measuring the incorporation of [^3^H]thymidine into cellular DNA as described [85]. The coupling of TGF-β to p3TP- and BRE-luciferase reporter gene expression was undertaken as described previously [42]. Likewise, the ability of TGF-β1 or c-Abl to impact either the expression or activity of various intracellular effector molecules was determined by immunoblotting as described [42]. Antibodies used herein were as follows: *(a)* anti-p21 (1:500; Santa Cruz Biotechnology, Santa Cruz, CA); *(b)* anti-p53 (1:500; EDM Millipore, Billerica, MA); *(c)* anti-c-Abl (1:500; BD Biosciences, San Jose, CA); *(d)* anti-MMP-9 (1:500; Sigma); *(e)* anti-phospho-Smad1/5/8 (1:500; Cell Signaling Technologies); *(f)* anti-phospho-Smad2 (1:1000; Cell Signaling Technologies); *(g)* anti-phospho-Smad3 (1:1000:Cell Signaling Technologies); *(h)* anti-Smad2/3 (1:1000;Cell Signaling Technologies); and *(i)* anti-β-actin (1:1000; Sigma-Aldrich).

### 3-dimensional (3D) culture and β-galactosidase staining

NMuMG or 4T1 derivatives (7,500 cells/well) were cultured onto Cultrex cushions (150 ml; Trevigen, Gaithersburg, MD) housed in 48-well plates that contained complete media supplemented with 5% Cultrex. Where indicated, the Cultrex cushions were rendered biomechanically rigid by the inclusion of type I collagen (3 mg/ml; BD Biosciences), and cells propagated under compliant or rigid 3D-culture conditions were incubated in the absence or presence of TGF-β1 (5 ng/ml) as indicated. Differences in organoid growth were monitored by bright-field microscopy and quantified using Image J64 (version 1.46), while variations in β-galactosidase expression were detected and visualized using the Beta-Galactosidase Staining Kit (Mirus Bio LLC, Madison, WI) according to the manufacturer's instructions. Additionally, 4T1 derivatives (400,000 cells/well) were also cultured for 60 hr on Cultrex cushions (400 μl) housed in 6-well plates in the absence or presence of the TβRI inhibitor, SB431542 (10 μM) as indicated. Afterward, the resulting organoids were using the Cultrex 3D-culture cell harvesting kit (3448-020-K; Trevigen) according to the manufacturer's recommendations, at which point total RNA was isolated and purified as described above.

### Tumor studies

4T1 cells engineered to express empty vector (*i.e.*, Scram), CST-Abl, or CST-Abl in combination with shRNA against p21 (*i.e.*, CST-Abl/sh21) were engrafted into the 4^th^ inguinal mammary fat pad (10,000 cells/injection) of syngeneic 6–8 week old female Balb/C mice (Jackson Laboratories). Primary 4T1 tumor growth was monitored as described [27]. Upon completion of the studies, the injected mammary fat pads and primary 4T1 tumors were excised and weighed between days 21–28, and again on day 59 post-inoculation. All animal studies were performed according to animal protocol procedures approved by the Institutional Animal Care and Use Committee of the University of Colorado.

### DNA damage and survival assays

Parental (*i.e.*, GFP) NMuMG cells, as well as parental (*i.e.*, GFP) or CST-Abl-expressing 4T1 cells were cultured and allowed to adhere overnight in 48-well plates (20,000 cells/well), at which point they were exposed to 6-thioguanine (60 μM; Sigma-Aldrich) for 72 h at 37°C. Afterward, the surviving cells were *(i)* washed twice in ice-cold in PBS; *(ii)* immediately fixed in 95% ethanol for 60 min at room temperature; *(iii)* stained with crystal violet (BD Biosciences) for 30 min; and *(iv)* destained with deionized H_2_O. Afterward, the extent of cell survival relative to diluent treated cells was quantified by imaging densitometry of stained tissue culture wells using the Image J (version 1.46).

### RNA interference studies

CST-Abl-expressing 4T1 cells lacking expression of p53 were generated using SMARTpool siRNAs (50 nM) according to the manufacturer's recommendations (Dharmacon, Lafayette, CO) and as previously described [86]}. Ninety-six hr post-transfection, the cells were harvested and total RNA was isolated to monitor the expression of p21 mRNA by semi-quantitative real-time PCR as described above. CST-Abl cell transfection efficiency was monitored by co-transfection with si*GLO* RISC-Free siRNA (Dharmacon), which was visualized using fluorescent microscopy.

### Statistical analyses

Statistical values were defined using an unpaired Student's *t-test* with a *P value* of < 0.05 considered significant.

## SUPPLEMENTARY MATERIALS TABLES FIGURES



## Abbreviations

3D: 3-dimensional
CML: chronic myelogenous leukemia
CST: constitutively-active
E-cad: epithelial cadherin
EMT: epithelial mesenchymal transition
MEC: mammary epithelial cell
MET: mesenchymal-epithelial transition
MMP: matrix metalloproteinase
TGF-β: transforming growth factor-β
TNBC: triple-negative breast cancer.
