# Supplementary material for: c-Abl inhibits breast cancer tumorigenesis through reactivation of p53-mediated p21 expression
Source: Oncotarget. 2016 Sep 8;7(45):72777–94. doi: 10.18632/oncotarget.11909 (PMC5340126; doi:10.18632/oncotarget.11909)
Supplement: Supplementary file 1 [file oncotarget-07-72777-s001.pdf]

# c-Abl inhibits breast cancer tumorigenesis through reactivation of p53-mediated p21 expression

## Supplementary Materials

## SUPPLEMENTARY MATERIALS AND METHODS

### MMP-9 vectors and cell lines

An autoactivating MMP-9 mutant was synthesized by site-directed mutagenesis of the murine MMP-9 cDNA (Open Biosystems) to replace $Gly^{100}$ with Leu (*i.e.,* G100L-MMP-9) using the QuikChange site-directed mutagenesis kit (Agilent Technologies, Santa Clara, CA) as described previously [1]. After the full-length G100L-MMP-9 cDNA was subcloned into a pBabe-puro retroviral expression vector, control (*i.e.,* empty vector) and G100L-MMP-9 retroviral supernatants were produced and transduced into parental and CST-Abl-expressing 4T1 cells as described [2]. Polyclonal populations of control and G100L-MMP-9-expressing 4T1 derivatives were isolated following puromycin (5 μg/ml) selection for 2 weeks. Likewise, polyclonal populations of 4T1 cells lacking MMP-9 expression were generated by their lentiviral-mediated transduction of shRNAs against MMP-9 (cat# RMM4534-NM_013599, cloneTRCN0000031231; Open Biosystems) as described [3]. In both cases, the extent to which MMP-9 expression and activity were altered was determined by the MMP-9 fluorimetric assays, and by immunoblotting analyses as described [2].

### Invasion assays

The ability of MMP-9 expression and activity to impact the invasiveness of parental (*i.e.,* empty vector) and CST-Abl-expressing 4T1 cells was determined by measuring their invasion through Matrigel-coated (diluted 1:25 in serum-free medium; BD Biosciences) modified Boyden chambers as described [2].

### MMP-9 tumor studies

4T1 cells engineered to express either empty vector (*i.e.,* Scram), shRNA against MMP-9 (*i.e.,* shMMP-9), autoactivating G100L-MMP-9 (*i.e.,* CaMMP9), CST-Abl, or CST-Abl in combination with CaMMP9 were engrafted into the 4th inguinal mammary fat pad (10,000 cells/injection) of syngeneic 6–8 week old female Balb/C mice (Jackson Laboratories). Primary 4T1 tumor growth was monitored over a span of 28 days as described [2]. All animal studies were performed according to animal protocol procedures approved by the Institutional Animal Care and Use Committee of the University of Colorado.

### UHCMC TMA immunohistochemical analyses

Deidentified human breast tissue microarrays were obtained through University Hospitals Case Medical Center (UHCMC)/Case Comprehensive Cancer Center (CCCC) and were previously processed and evaluated for ER, PR, and HER2 expression (IRB Protocol 01-13-43C and CASE 7114). A total of 157 tumor samples were evaluated and represented the following receptor-defined subtypes: ER + /PR + /HER2 – ($n$ = 49), ER – /PR – /HER2 + ($n$ = 29), ER + /PR + /HER2+ ($n$ = 20), ER – /PR – /HER2 – ($n$ = 47), and other patients ($n$ = 12). Additional clinicopathologic characteristics are described elsewhere [4]. Immunohistochemical (IHC) staining of c-Abl and p53 in formalin-fixed paraffin-embedded normal and tumor tissues was performed using anti-c-Abl antibody (Abcam Inc. Cat. # ab15130) and the CONFIRM p53 clone D0-7 (Ventana Medical Systems Inc. Cat. #790-2912), respectively. Slides were baked at 60°C, dewaxed, and rehydrated. Epitopes were retrieved by heating the slides for 20 min using either the Bond Epitope Retrieval 1 for c-Abl (citrate–based solution, pH 6.0; Leica Biosystems) or Cell Conditioning 1 for p53 (ethylenediaminetetraacetic acid-based solution, pH 9.0, Ventana Medical Systems Inc.), at which point all slides were treated for an additional 5 min treatment with 3% hydrogen peroxide to inactivate endogenous peroxidase. Afterward, the slides were incubated with either the anti-c-Abl rabbit polyclonal antibody (2 μg/mL) or anti-p53 antibody (ready-to-use reagent) for 15 min, and subsequently were developed using a polymer based detection system and counterstained with hematoxylin. All tissue slides were evaluated and scored by a board-certified pathologist. c-Abl immunostaining was quantified by signal intensity on a 3+ scale, where samples were placed into low (1) and high (2–3+) groups to facilitate statistical analyses. Positive p53 staining was indicative of breast cancers that expressed mutant p53. Statistical comparisons analyzing the probability of patient survival outcomes between groups were performed using the log-rank test.

# REFERENCES


1. Galliher AJ, Schiemann WP. Src phosphorylates Tyr284 in TGF-β type II receptor and regulates TGF-β stimulation of p38 MAPK during breast cancer cell proliferation and invasion. Cancer Res. 2007; 67:3752–8.
2. Allington TM, Galliher-Beckley AJ, Schiemann WP. Activated Abl kinase inhibits oncogenic transforming growth factor-β signaling and tumorigenesis in mammary tumors. FASEB J. 2009; 23:4231–43.
3. Taylor MA, Amin J, Kirschmann DA, Schiemann WP. Lysyl oxidase contributes to mechanotransduction-mediated regulation of transforming growth factor-β signaling in breast cancer cells. Neoplasia. 2011; 13:406–18.
4. Sizemore ST, Sizemore GM, Booth CN, Thompson CL, Silverman P, Bebek G, Abdul-Karim FW, Avril S, Keri RA. Hypomethylation of the MMP7 promoter and increased expression of MMP7 distinguishes the basal-like breast cancer subtype from other triple-negative tumors. Breast Cancer Res Treat. 2014; 146:25–40.


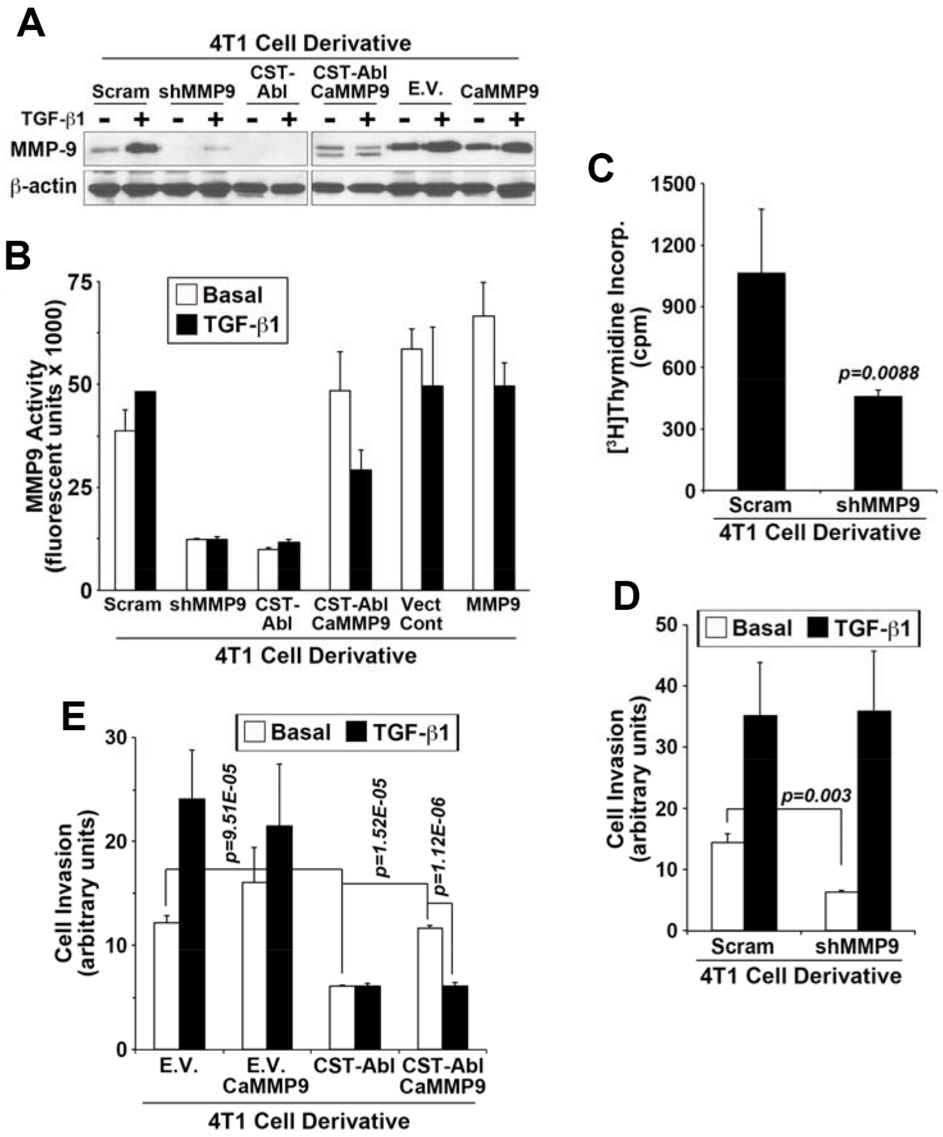


**Supplemental Figure S1: Differential requirement for MMP-9 activity in regulating 4T1 cell proliferation and invasion.** (**A** and **B**) 4T1 cell derivatives were incubated in the absence or presence of TGF-β1 (5 ng/ml) for 24 h. Afterwards, alterations in MMP-9 expression was measured by immunoblotting (A), while differences in MMP-9 activity was assessed by fluorimetric assays (B). (**C**) DNA synthesis rates in parental (*i.e.,* Scram) and MMP-9-deficient 4T1 cells were determined by [$^3$H]thymidine incorporation assays, which implicated MMP-9 as a significant promoter of 4T1 cell proliferation. (**D**) The invasiveness of parental (*i.e.,* Scram) and MMP-9-deficient (shMMP9) 4T1 cells incubated in the absence or presence of TGF-β1 (5 ng/ml) determined using Modified Boyden chamber assays, which implicated MMP-9 as a significant mediator of basal 4T1 cell invasion, but not that stimulated by TGF-β. (**E**) Expression of an autoactivated MMP-9 mutant (CaMMP9) failed to overcome the inhibitory activities of CST-Abl on 4T1 cell invasion. Data are mean (± SE) of 2 experiments completed in duplicates. *E.V.,* empty vector. *Scram,* scrambled shRNA.

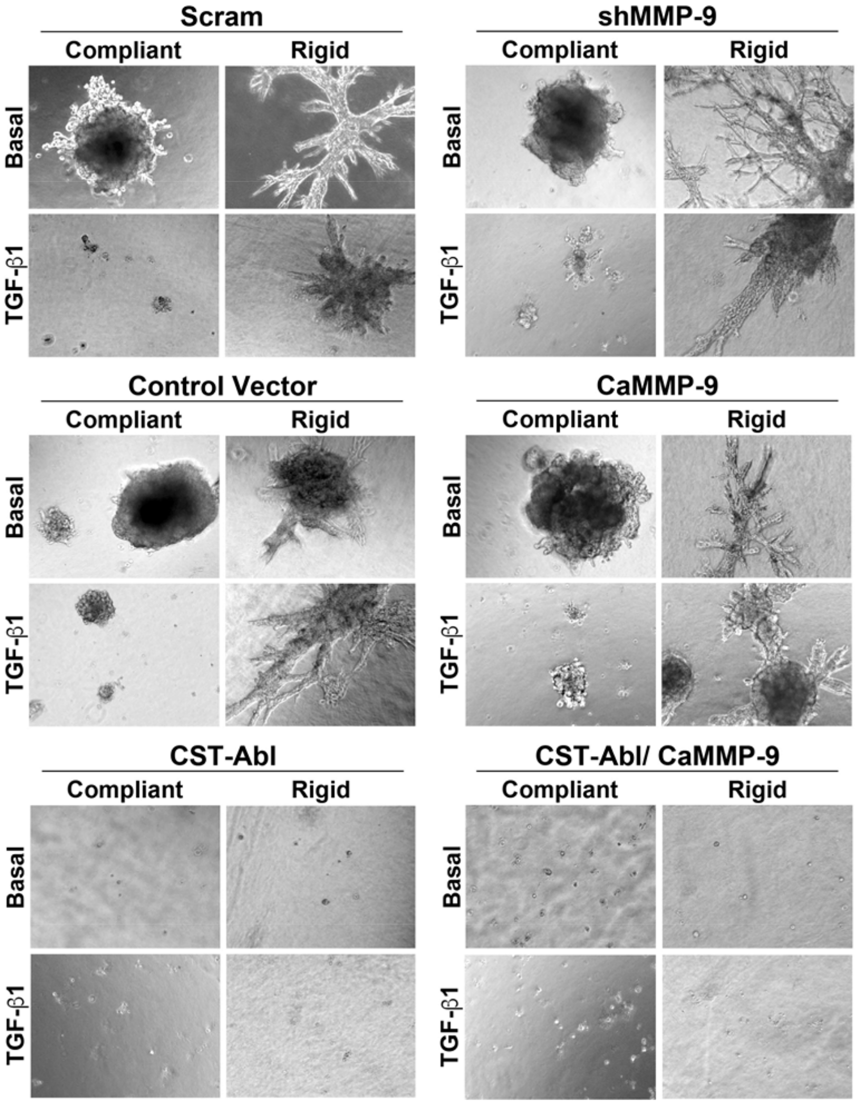


**Supplemental Figure S2: Increased or decreased expression and activity of MMP-9 failed to impact the morphology of 4T1 organoids propagated for 10 days in compliant or rigid (2 mg/ml type I collagen) 3D-cultures as indicated.** Photomicrographs are representative of 3 independent experiments. *CaMMP-9*, autoactivating MMP-9 mutant.

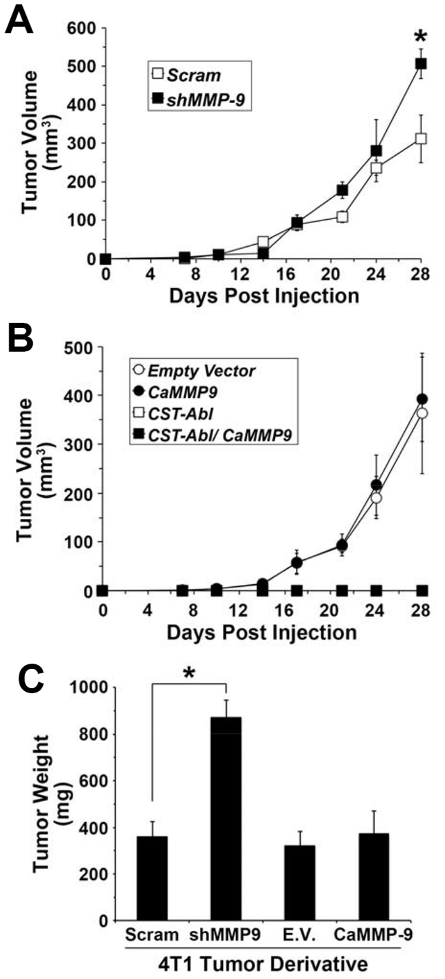


**Supplemental Figure S3: Restoring MMP-9 expression and activity in CST-Abl-expressing 4T1 cells fails to rescue their tumorigenicity.** (**A** and **B**) Parental (*i.e.,* Scram), MMP-9-deficient (shMMP9), CaMMP9-, CST-Abl-, or CST-Abl/CaMMP9-expressing 4T1 cells (10,000 cells/mouse) were engrafted into the fat pads of female Balb/C mice. Tumor growth was monitored using digital calipers on the indicated days post-engraftment. Data are the mean (± SE; $n = 10$) tumor volumes. (C) Primary tumors from the indicated cohorts were excised and weighed at the time of sacrifice. *$P < 0.05$.

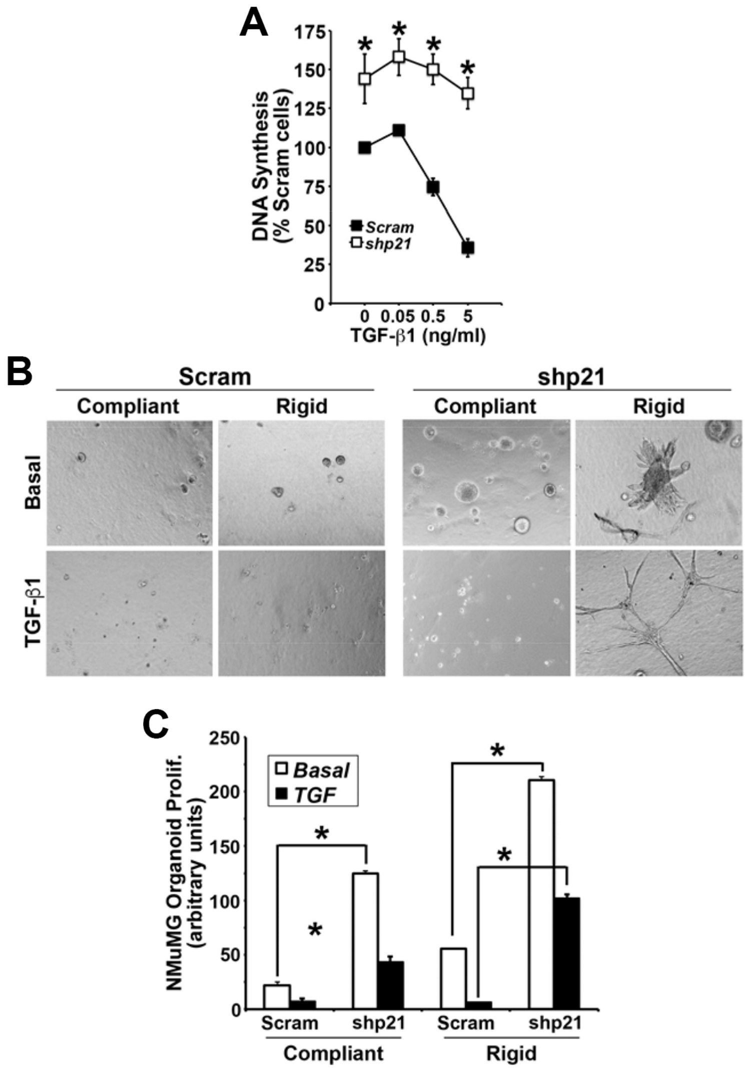


**Supplemental Figure S4: p21 expression is essential for TGF-β-mediated growth arrest and induction of apoptosis in rigid microenvironments.** (**A**) Parental (*i.e.,* Scram) and p21-deficient NMuMG cells were incubated in absence or presence of increasing concentrations of TGF-β1 (0→5 ng/ml) as indicated, and differences in DNA synthesis rates were determined by [$^3$H]thymidine incorporation assays. Data are the mean (± SE) of 3 independent experiments. *$P < 0.05$. (**B** and **C**) Parental (*i.e.,* Scram) and p21-deficient NMuMG cells were propagated in compliant or rigid (3 mg/ml type I collagen) 3D-cultures for 5 d. Photomicrographs are representative of 3 independent experiments (**B**), while the extent of NMuMG organoid proliferation was quantified using Image J (**C**). Date are the mean (± SE) of 3 independent experiments. *$P < 0.05$.

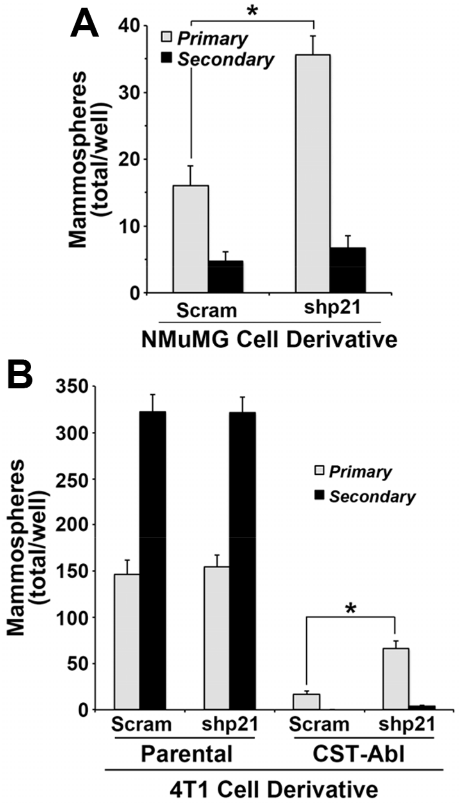


**Supplemental Figure S5: p21 regulates mammosphere formation in normal and malignant mammary epithelial cells.** (**A**) Parental (*i.e.,* Scram) and p21-deficient NMuMG cells were propagated in low-adherent plates to quantify primary and secondary mammosphere formation. Data are the mean (± SE) of 3 independent experiments. **P* < 0.01. (**B**) Parental (*i.e.,* Scram) and CST-Abl-expressing 4T1 cells were rendered deficient in p21 expression (shp21) and the impact of this manipulation on primary and secondary mammosphere formation was quantified. Data are the mean (±SE) of 3 independent experiments. **P* < 0.001.

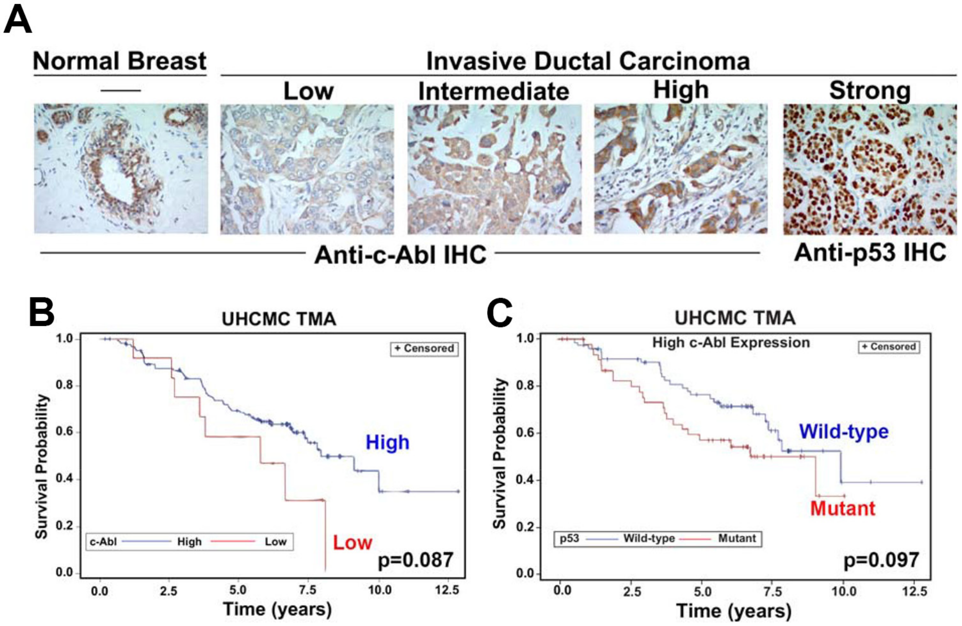


**Supplemental Figure S6: Mutant p53 expression trends to promote the oncogenic activities of c-Abl.** (**A**) Representative staining of c-Abl expression in normal and invasive ductal carcinoma (IDC) samples and representative positive staining for p53 expression in IDC sample. (**B**) Kaplan-Meier curve demonstrating the probability of survival for the UHCMC TMA patient population stratified by c-Abl staining. High c-Abl expression trends to associate with better probability of survival. (**C**) Kaplan-Meier curve demonstrating the probability of survival for the UHCMC TMA patient population that retain high c-Abl expression stratified by the absence or presence of mutant p53 expression. Mutant p53 expressing patients trended to have reduced probabilities of survival when high c-Abl expression is retained.

**Supplemental Table S1: SAGE anatomic viewer results**

| Tissue | Normal* | Cancer* |
|---|---|---|
| Brain | < 2 | 2 to 3 |
| Retina | 2 to 3 | 4 to 7 |
| Thyroid | < 2 | < 2 |
| Lung | < 2 | 2 to 3 |
| Heart | < 2 | N.A. |
| Breast & Breast Cell Types | 32 to 63 | 2 to 3 |
| Stomach | < 2 | < 2 |
| Pancreas | < 2 | < 2 |
| Liver | < 2 | < 2 |
| Kidney | 4 to 7 | < 2 |
| Colon | 2 to 3 | 2 to 3 |
| Peritoneum | 4 to 7 | < 2 |
| Spinal Cord | 4 to 7 | No data |
| Ovary | No data | 2 to 3 |
| Placenta | < 2 | N.A. |
| Prostate | 4 to 7 | < 2 |
| Bone Marrow | 2 to 3 | 4 to 7 |
| Cartilage | No data | 2 to 3 |
| Muscle | < 2 | < 2 |
| Skin | 2 to 3 | 2 to 3 |
| Lymph Node | < 2 | < 2 |
| White Blood Cells | < 2 | No data |
| Vascular | 4 to 7 | No data |

*SAGE tags per 200,000. Data derived from SAGE Genie tool Cancer Genome Array Project (http://cgap.nci.nih.gov).

## Supplemental Table S2: Semi-quantitative real-time PCR oligonucleotide sequences

**Mouse**

| Target | Application | Sequence (5′ to 3′) |
|---|---|---|
| p21 | PCR-Sense | 5′-TCCAGGAGGCCCGAGAACGG |
| p21 | PCR-Antisense | 5′-TAGAAATCTGTCAGGCTGGTCTGCC |
| Sox2 | PCR-Sense | 5′-TAGAGCTAGACTCCGGGCGATGA |
| Sox2 | PCR-Antisense | 5′-TTGCCTTAAACAAGACCACGAAA |
| Klf4 | PCR-Sense | 5′-GCGAACTCACACAGGCGAGAAACC |
| Klf4 | PCR-Antisense | 5′-TCGCTTCCTCTTCCTCCGACACA |
| GAPDH | PCR-Sense | 5′-CAACTTTGGCATTGTGAAAGGGCTC |
| GAPDH | PCR-Antisense | 5′-GCAGGGATGATGTTCTGGGCAGC |

**Human**

| Target | Application | Sequence (5′ to 3′) |
|---|---|---|
| c-Abl | PCR-Sense | 5′-CTCCTGGACCTTGACAGAGC |
| c-Abl | PCR-Antisense | 5′-AACCGCATAAAACGATCCAG |
| p53 | PCR-Sense | 5′- CCCAAGCAATGGATGATTTGA |
| p53 | PCR-Antisense | 5′-GGCATTCTGGGAGCTTCATCT |
| GAPDH | PCR-Sense | 5′-TGCACCACCAACTGCTTAGC |
| GAPDH | PCR-Antisense | 5′-GGATGGACTGTGGTCATGAG |
